# The complete chloroplast genome sequence and phylogenetic analysis of *Tragopogon pratensis* L. (Asteraceae)

**DOI:** 10.1080/23802359.2024.2384578

**Published:** 2024-08-15

**Authors:** Xin-xue Zhang, Jianhu Zhang, Peng Wan, Hua Liu

**Affiliations:** a Guyuan Branch, Ningxia Academy of Agricultural and Forestry Sciences, Guyuan, China; b Institute of Forestry and Grassland Ecology, Ningxia Academy of Agricultural and Forestry Sciences, Yinchuan, China

**Keywords:** *Tragopogon pratensis*, chloroplast genome, phylogenetic analysis

## Abstract

Currently, the phylogenetic relationships of *Tragopogon pratensis* Linnaeus (1753) remain unclear. This study presents the first report on the complete chloroplast genome of *T. pratensis*, which is a quadripartite structure with a length of 153,002 bp and containing a large single copy (LSC, 84,225 bp) region, a small single copy (SSC, 18,407 bp) region, a pair of inverted repeats (IR, 25,185 bp) regions. A total of 134 genes are identified, including 87 protein-coding genes, 8 rRNA genes, 37 tRNA genes, and 2 pseudogenes. Phylogenetic analysis revealed that *T. pratensis* is most closely related to *T. dubius* and *that Tragopogon* is a monophyletic genus.

## Introduction

*Tragopogon pratensis* Linnaeus (1753), which belongs to the tribe Lactuceae in the family Asteraceae, is typically a biennial species but occasionally exhibits a perennial habit. It is native to Europe and North Africa and is commonly found on roadside verges, dikes, and ruderal places with varying soil types ranging from clay to sand and gravel (Qi et al. 1996, Mölken et al. 2005). *Tragopogon pratensis* is a monocarpic grassland species that does not propagate vegetatively nor does it form a persistent seed bank. It flowers from late May until the end of July and dies after producing seeds (Jorritsma-Wienk et al. 2007). Although *T. pratensis* is a diploid species, its phylogenetic relationships with *T. pratensis* remain unclear. Therefore, our study aims to confirm the phylogenetic relationships by reporting its complete chloroplast genome for the first time, thereby offering novel insights into its phylogenetic relationships.

## Materials and methods

**Figure 1.**
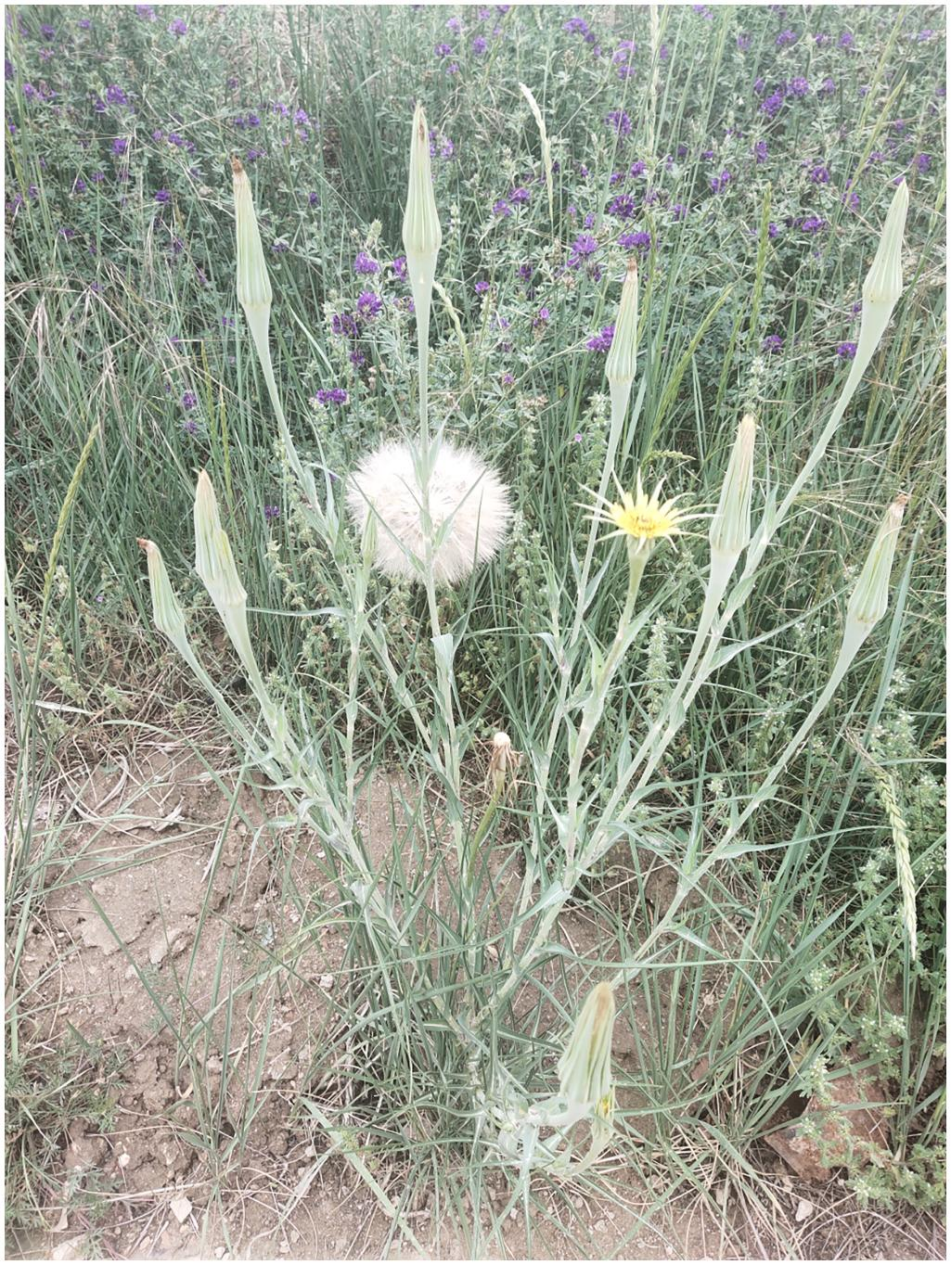
The morphological characteristics of *T. pratensis*. The photographs were taken by Xin-xue Zhang at guyuan (106°17′ E, 35°72′N), Ningxia, China.

Fresh young leaves of *T. pratensis* are collected from the wild at Guyuan (106°17′ E, 35°72′ N), Ningxia, China (Figure 1), and they are dried using silica gel. Voucher specimens are deposited at Guyuan Branch, Ningxia Academy of Agricultural and Forestry Sciences (voucher specimens: zxx001, contact person: Xin-xue Zhang 476102527@qq.com). Genomic DNA is extracted by the modified cetyltrimethylammonium bromide (CTAB) method (Doyle and Doyle 1987) which is from approximately 20 mg of silica gel-dried leaf tissues. Subsequently, a paired-end (150 bp) DNA library with an insert size of approximately 400 bp is constructed using a TruSeq DNA Sample Prep Kit (Illumina, Inc., San Diego, CA, USA). The library is then sequenced on the Illumina Xten platform at BioMarker (Beijing, China), generating approximately 67.2 Mb reads and 10.0 Gb bases.

The complete chloroplast genome of *T. pratensis* is *de novo* assembled using GetOrganelle toolkit (Jin et al. 2020) with the default parameters, while the complete chloroplast genome of *T. dubius* (OR840963.1)(Unpublished) is selected as a reference genome. To evaluate sequencing depth and coverage, BWA (Li and Durbin 2009) is employed to map the reads to reference sequence, and the depth and coverage are examined by SAMtools (Li and Durbin 2009). The high coverage and mean depth are 99.89% and 3948.3×, respectively (Figure S1). Subsequently, annotation is carried out on CPGAVAS2 online (Shi et al. 2019), followed by the generation of a circular map of *T. pratensis* using the CPGView online tool (Liu et al. 2023).

To determine the phylogenetic relationships of *T. pratensis*, we select 31 individuals from the tribe Lactuceae (Asteraceae) as ingroups, *Calendula officinalis* (OP161555.1, Unpublished) and *Blumea aromatica* (ON470223.1, Unpublished) are used as outgroups. Additionally, to test the phylogenetic relationships from different regions, we employ three datasets: (1) the complete chloroplast genome, (2) coding sequences (CDS), (3) concatenated from *mat*K, *rbc*L, and *trn*H-*psb*A. The sequences are aligned using the MAFFT program (Katoh and Standley 2013). Subsequently, the phylogenetic tree is generated using IQ-TREE (Nguyen et al. 2015) through maximum likelihood (ML) method and the best-fit model of TVM + F + I + R4. In the phylogenetic tree analysis, 1000 replicates are performed to establish robust statistical support. Additionally, the SH-aLRT test (Chen et al. 2022) is employed to assess the branch support.

## Results

**Figure 2.**
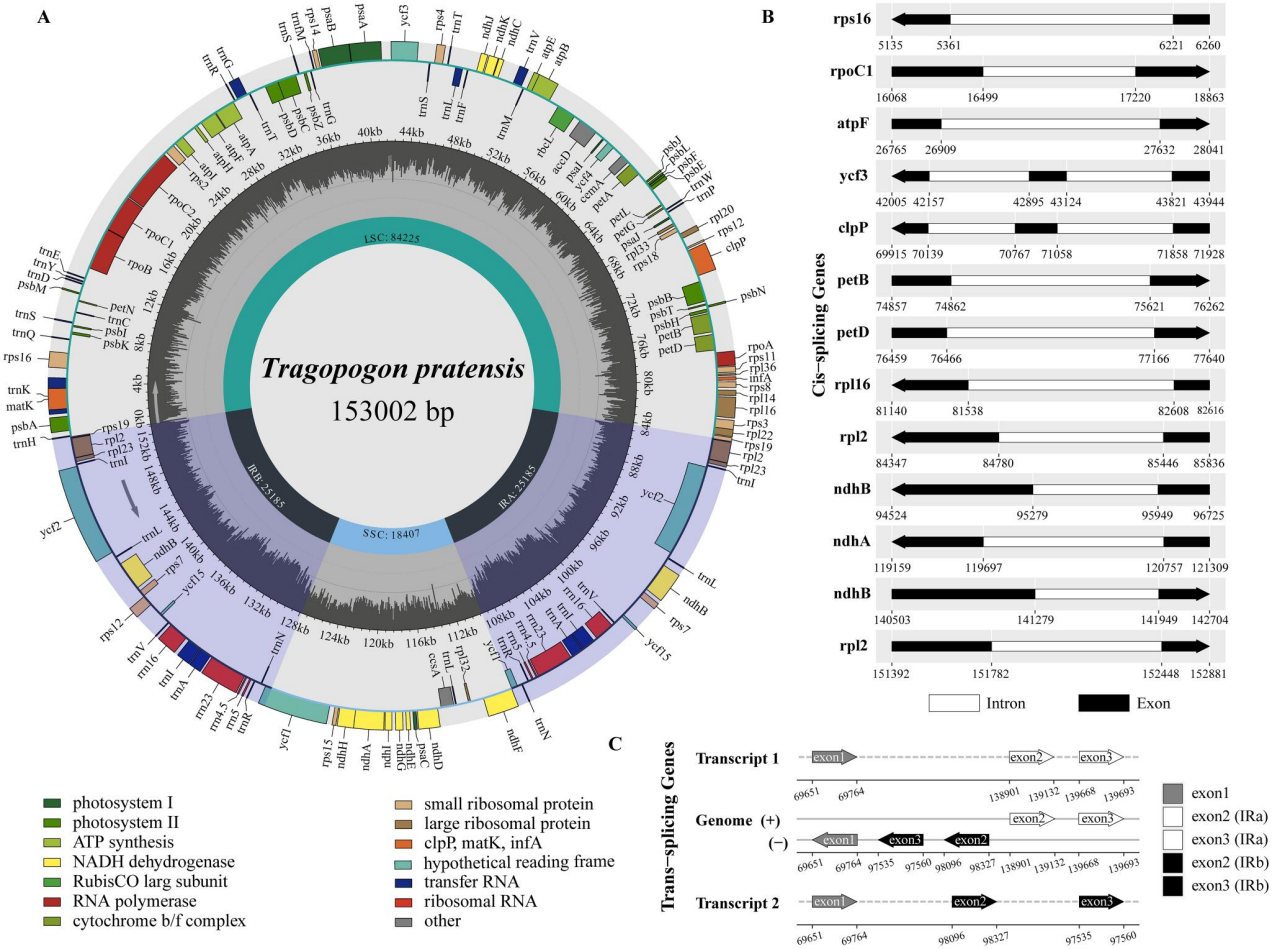
Schematic map of *T. pratensis* complete chloroplast genome (a), genes shown inside and outside the circle are transcribed clockwise and counterclockwise, respectively. Genes belonging to different functional groups are color coded. The GC and at contents are indicated by the dark grey and light grey colors in the inner circle, respectively. The structure of cis-splicing genes (B). The trans-splicing gene *rps*12 (C).

The complete chloroplast genome of *T. pratensis* exhibited a typical quadripartite structure, with 153,002 bp and 37.7% GC content. It consisted of a large single copy (LSC, 84,225 bp) region, a small single copy (SSC, 18,407 bp) region, a pair of inverted repeats (IR, 25,185 bp) regions (Figure 2(A)). A total of 134 genes are annotated in the complete chloroplast genome of *T. pratensis*, comprising 87 protein-coding genes, 8 rRNA genes, 37 tRNA genes, and 2 pseudogenes. In addition, among these genes, nine unique protein protein-coding (*rps*16, *rpo*C1, *atp*F, *pet*B, *pet*D, *rpl*16, *rpl*2, *ndh*B, *ndh*A) contained only one intron each, but *ycf*3 and *clp*P genes with two introns (Figure 2(B)). Furthermore, *rps*12 gene was identified as trans-spliced gene, and two duplicated 3′ end exons in IR regions and one 5′ end exon in the LSC region (Figure 2(C)).

**Figure 3.**
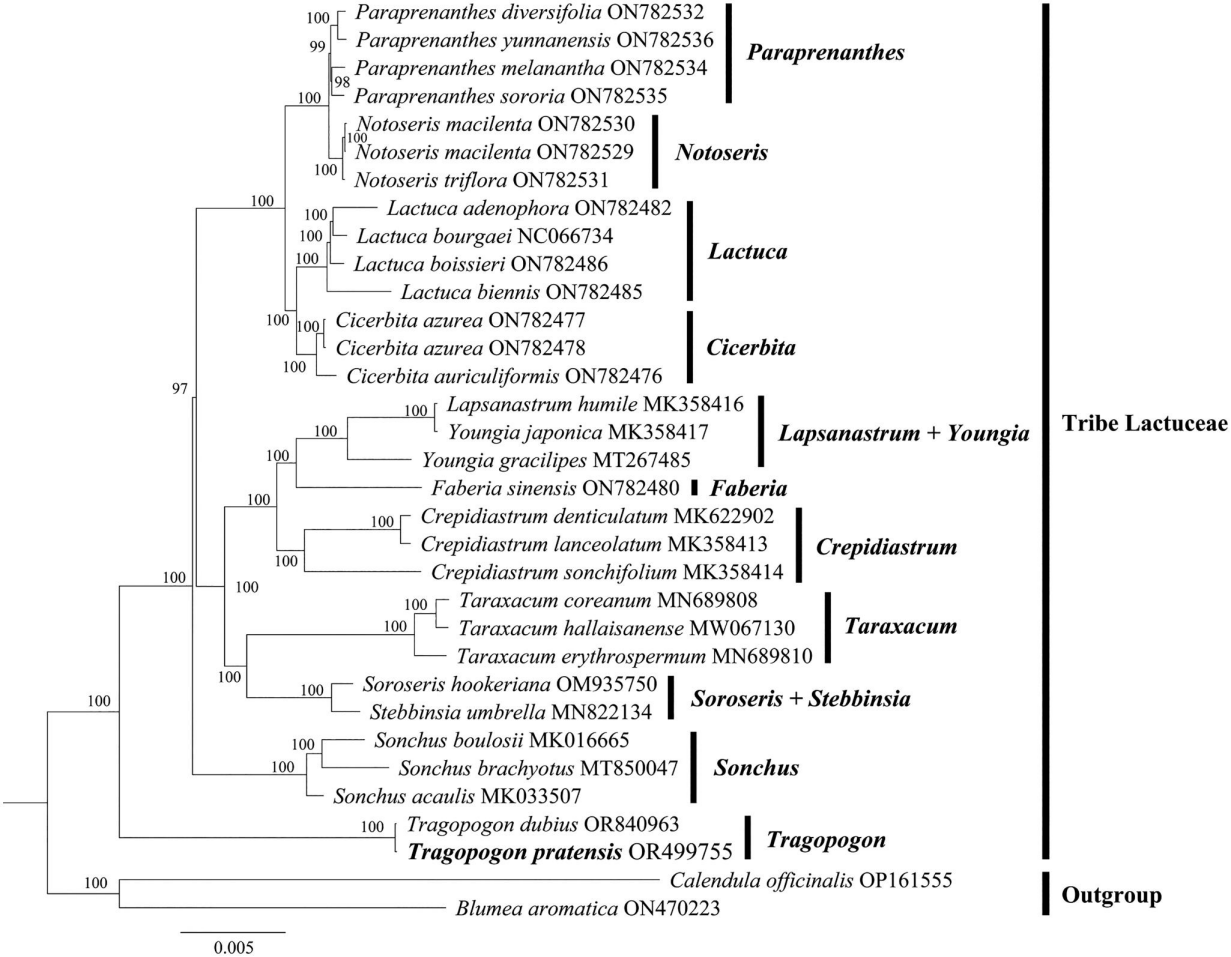
Maximum likelihood (ML) phylogenetic tree based on complete chloroplast genome. The following sequences were used: *Tragopogon pratensis* OR499755 (this study), *cicerbita auriculiformis* ON782476 (Unpublished), *C. azurea* ON782477 (Unpublished), *C. azurea* ON782478 (Unpublished), *crepidiastrum denticulatum* MK622902 (Do et al. 2019), *C. lanceolatum* MK358413 (Nguyen et al. 2023), *C. sonchifolium* MK358414 (Unpublished), *faberia sinensis* ON782480 (Unpublished), *lactuca adenophora* ON782482 (Unpublished), *L. biennis* ON782485 (Unpublished), *L. boissieri* ON782486 (Unpublished), *L. bourgaei* NC066734 (Unpublished), *lapsanastrum humile* MK358416 (Unpublished), *notoseris macilenta* ON782529 (Unpublished), *N. macilenta* ON782530 (Unpublished), *N. triflora* ON782531 (Unpublished), *paraprenanthes diversifolia* ON782532 (Unpublished), *P. melanantha* ON782534 (Unpublished), *P. sororia* ON782535 (Unpublished), *P. yunnanensis* ON782536 (Unpublished), *sonchus acaulis* MK033507 (Cho et al. 2019), *S. boulosii* MK016665 (Kim et al. 2019), *S. brachyotus* MT850047 (Wang et al. 2021), *soroseris hookeriana* OM935750 (Unpublished), *stebbinsia umbrella* MN822134 (Lv et al. 2020), *taraxacum coreanum* MN689808 (Lee et al. 2020), *T. erythrospermum* MN689810 (Lee et al. 2020), *T. hallaisanense* MW067130 (Lee et al. 2021), *T. dubius* OR840963 (Unpublished), *youngia gracilipes* MT267485 (Unpublished), *Y. japonica* MK358417 (Unpublished), *blumea aromatica* ON470223 (Unpublished), *calendula officinalis* OP161555 (Unpublished).

The phylogenetic trees exhibited different topologies which were constructed from three datasets. Phylogenetic analysis of the complete chloroplast genome, revealed that *T. pratensis* is most closely relative to *T. dubius* (BS = 100%), and it clustered within a single clade with *T. dubius*, indicating the monophyly of *Tragopogon* with a high bootstrap value (BS = 100%) (Figure 3). Additionally, the congruent conclusion is supported by the phylogenetic tree constructed using two datasets (Figure S2), respectively, where *Tragopogon* formed a monophyletic clade and *T. pratensis* is identified as the closest relative to *T. dubius* (BS = 100%).

## Discussion and conclusion

In this study, the complete chloroplast genome of *T. pratensis* is a quadripartite structure with 153,002 bp, containing a large single copy (LSC) region, a small single copy (SSC) region, a pair of inverted repeats (IR) regions. This result is similar to other species in the tribe Lactuceae (Asteraceae), such as *Soroseris umbrella* and *Ixeris repens* (Lv et al. 2020; Lee et al. 2021). The present study ultimately furnishes evidence supporting the monophyly of *Tragopogon*, and the ITS data also suggested that *Tragopogon* was monophyletic (Soltis et al. 2023). Due to the limited availability of the complete chloroplast genome in the genus *Tragopogon*, further investigation is required to validate the monophyly of *Tragopogon*. Therefore, future studies should aim to increase the sample numbers to ensure the accuracy of its monophyletic.

## Supplementary Material

Supplementary_Figure.docx

Figure S2.pdf

## Data Availability

Data from this study are available in GenBank (OR499755.1), The associated BioProject, SRA, and BioSample numbers are PRJNA1028000, SRR26384696, SAMN37810819, respectively.
