# Supplementary material for: The complete chloroplast genome sequence and phylogenetic analysis of Tragopogon pratensis L. (Asteraceae)
Source: Mitochondrial DNA B Resour. 2024 Aug 15;9(8):1077–80. doi: 10.1080/23802359.2024.2384578 (PMC11328793; doi:10.1080/23802359.2024.2384578)
Supplement: Supplementary_Figure.docx [file TMDN_A_2384578_SM6945.docx]

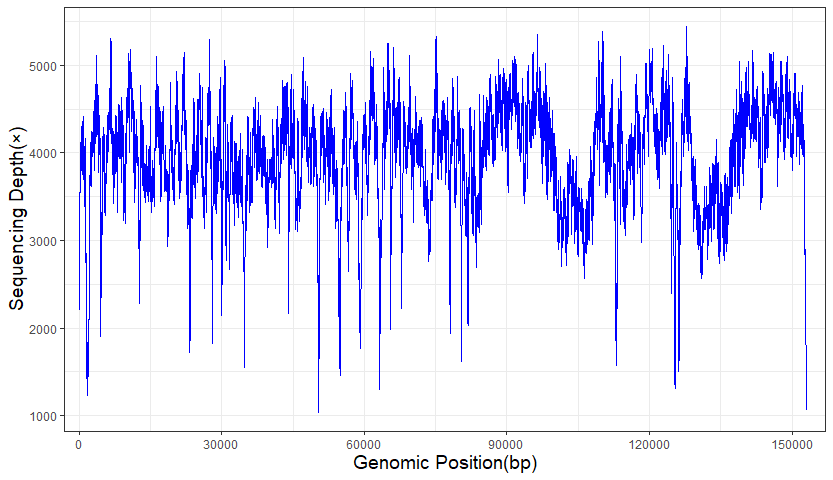


Figure S1 The sequencing depth results of *Tragopogon pratensis*. The average sequencing depth is 3948.3×, the maximum sequencing depth is 5441×, and the minimum sequencing depth is 1041×, respectively.
