# Supplementary material for: The complete chloroplast genome sequence and phylogenetic analysis of Tragopogon pratensis L. (Asteraceae)
Source: Mitochondrial DNA B Resour. 2024 Aug 15;9(8):1077–80. doi: 10.1080/23802359.2024.2384578 (PMC11328793; doi:10.1080/23802359.2024.2384578)

Figure S2 the phylogenetic tree from two datasets. (A) concatenated from *mat*K, *rbc*L, and t*rn*H-*psb*A, (B) coding sequences (CDS).

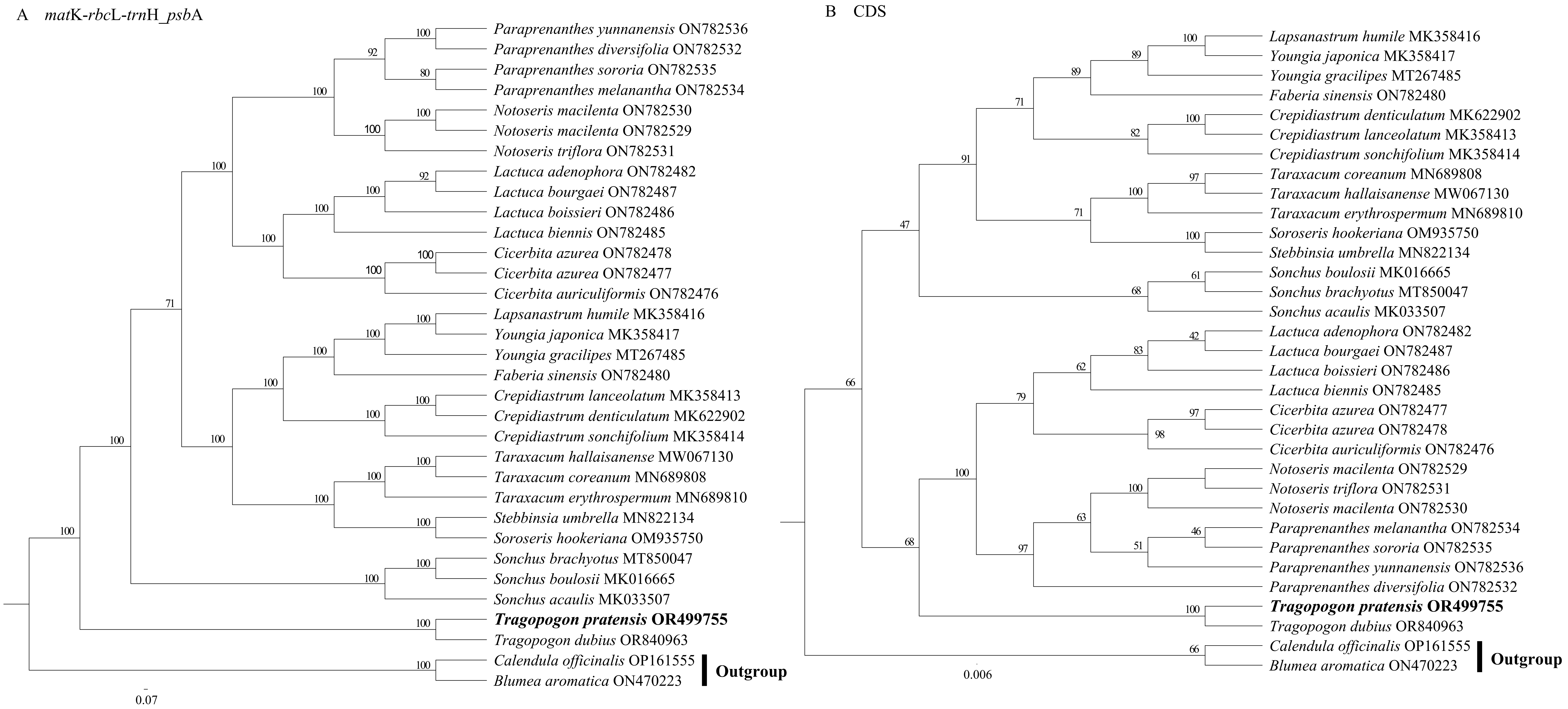

Supplement: Figure S2.pdf [file TMDN_A_2384578_SM6943.pdf]
